# Health Behaviors in Times of COVID: Different Sources of Support for Older Adults

**DOI:** 10.1093/geroni/igab046.2737

**Published:** 2021-12-17

**Authors:** Elizabeth Zambrano Garza, Theresa Pauly, Rachel Murphy, Wolfgang Linden, Maureen Ashe, Denis Gerstorf, Kenneth Madden, Christiane Hoppmann

**Affiliations:** 1 University of British Columbia, Vancouver, British Columbia, Canada; 2 University of Zurich, University of Zurich, Zurich, Switzerland; 3 University of British Columbia, University of British Columbia, British Columbia, Canada; 4 Humboldt University of Berlin, Humboldt University of Berlin, Brandenburg, Germany

## Abstract

Eating a nutritious diet reduces vulnerability to common chronic diseases. Yet, older adults struggle to meet nutritional guidelines; many have found it particularly challenging to access fresh foods such as fruits and vegetables during the pandemic. Thus, it is vital to better understand how older adults may recruit the help of close others to support healthy dietary intake. This COVID-19 study examines the role of support for promoting fruit and vegetable consumption in daily life. Ninety-seven older adults participated with a close other of their choice (62 % spouse; 38% non-spouse Mage partner1 = 72, SD = 5.26, Mage partner2 = 62, SD = 16.38). Both partners completed two daily questionnaires for 10 days. In the morning they reported their intentions for eating fruit and vegetables. In the evening, they noted their consumed fruit and vegetable servings, the extent to which this matched their intentions, and their partners support in doing so. Consistent with previous research, the older participants were, the more they consumed fruits and vegetables. On days when participants received more support from their partner, they were more successful at reaching their dietary goals. Interestingly, initial findings suggest that associations were stronger when support was provided from a non-spouse than if the support came from spouse. Follow-up analyses, with a larger sample, will further examine some of the underlying mechanisms so as to better understand the role of different kinds of support providers during the pandemic and shed light on who may be best suited to provide support.

